# Treatment of Pipkin type I fracture using safe surgical hip dislocation: A case report

**DOI:** 10.1002/ccr3.4147

**Published:** 2021-05-15

**Authors:** Paa Kwesi Baidoo, Kwasi Twumasi Baah Jnr, Anning Abu, Alex Osei Assim, Emmanuel Kafui Ayodeji, Senyo Gudugbe

**Affiliations:** ^1^ Directorate of Orthopedics and Trauma Komfo Anokye Teaching Hospital Kumasi Ghana; ^2^ Department of Surgery Komfo Anokye Teaching Hospital Kumasi Ghana

**Keywords:** complication, femoral head, fracture, outcome, Pipkin, surgical hip dislocation

## Abstract

We report the clinical and radiological outcomes of a 30‐year‐old female with femoral head fracture following a posterior hip dislocation. The patient was managed using safe surgical hip dislocation. She had a pain free range of motion of the hip at 1 year postinjury.

## Graphical abstract

Intraoperative images showing the femoral head fragment (A) and reduced fragment (B) which has been fixed with two 2.7 mm Herbert screws.

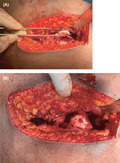

## 1 INTRODUCTION

Femoral head fractures are quite rare and may be associated with hip dislocations, femoral neck, and acetabular fractures.^1^, ^2^ The reported annual incidence is about 2 cases per million.^3^ Since Birkett^4^ first descriptions of these fractures in 1869, there have been many case reports. Pipkin^5^ in 1957 recommended a classification system for femoral head fractures, and this is widely used to this day. The classification divided these injuries into 4 types: type I involves the nonweight‐bearing part of the femoral head, type II affects the weight‐bearing part of the head of femur, type III may include either or both types I or II with femoral neck fracture, and type IV involves type I or II associated with acetabular fracture.^5^

Hip dislocations like any other joint dislocations are orthopedic emergencies and usually result from high energy injuries. The classical mechanism is from “dashboard injury” but may also result from sports injuries or fall from heights.^6^ Femoral head fractures have traditionally been known to have poor functional outcomes and high complication rates especially avascular necrosis (AVN) and post‐traumatic arthritis of the hip joint.^7^, ^8^, ^9^ It is well documented that early reduction should be done under anesthesia and adequate muscle relaxation, stabilization, and rigid fixation to achieve stable and congruent joint thereby reducing potential complication rate.^1^, ^6^ The best surgical approach on whether to fix or excise the femoral head fragment, however, remains controversial.^2^

Many approaches have been proposed for the fixation of these fractures, but the drawback is the limited exposure of the femoral head in all these approaches.^1^, ^8^ However, the current technique of safe surgical hip dislocation (SHD) as proposed by Ganz et al^10^ allows for complete exposure of the femoral head and acetabulum without interrupting the blood supply of the femoral head. We report our management of a patient with pipkin using the approach proposed by Ganz which conforms to the SCARE Criteria.^11^

## 2 CASE REPORT

**FIGURE 1:**
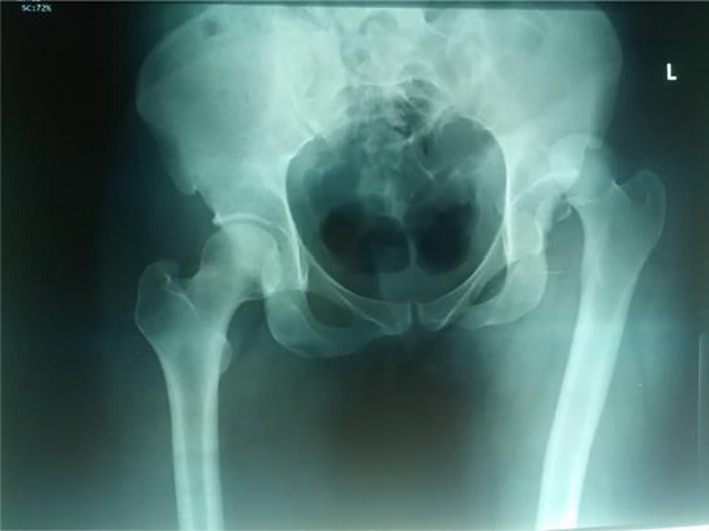
Preoperative pelvic X‐ray, AP, showing fracture dislocation of the left hip joint

Our patient is a 30‐year‐old Ghanaian female who was an unrestrained backseat passenger in a saloon car that was involved in a head‐on collision. The patient lost consciousness that lasted for about an hour after the accident. At presentation at the emergency department of the hospital, she had a Glasgow coma score (GCS) of 15/15. The patient sustained frontal scalp hematoma and multiple lacerations on the left lower limb. Her left lower limb was shortened, flexed at the hip, adducted, and internally rotated. Computerized tomography (CT) scan of the brain was normal, and a pelvic X‐ray showed posterior dislocation of the left hip associated with femoral head fracture (Figure 1). A diagnosed of posterior dislocation of the left hip (Pipkin type 1) was made.

**FIGURE 2:**
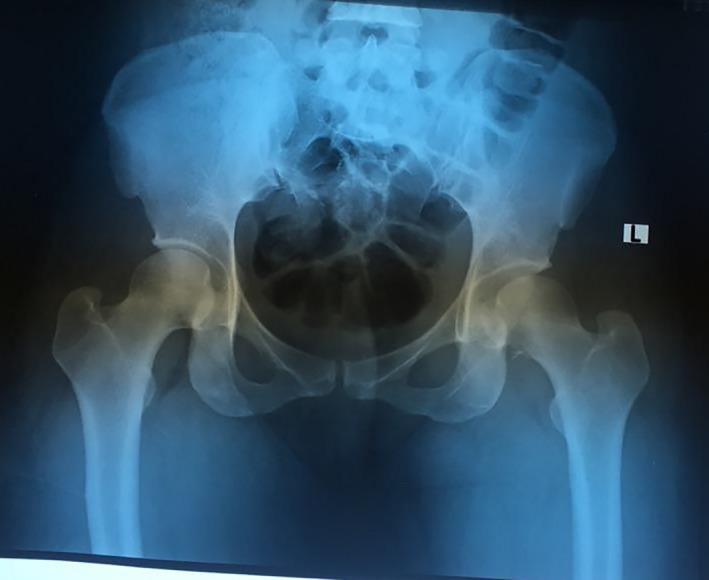
Postreduction pelvic X‐rays showing widening of the left hip joint and retained fragment of the femoral head

**FIGURE 3:**
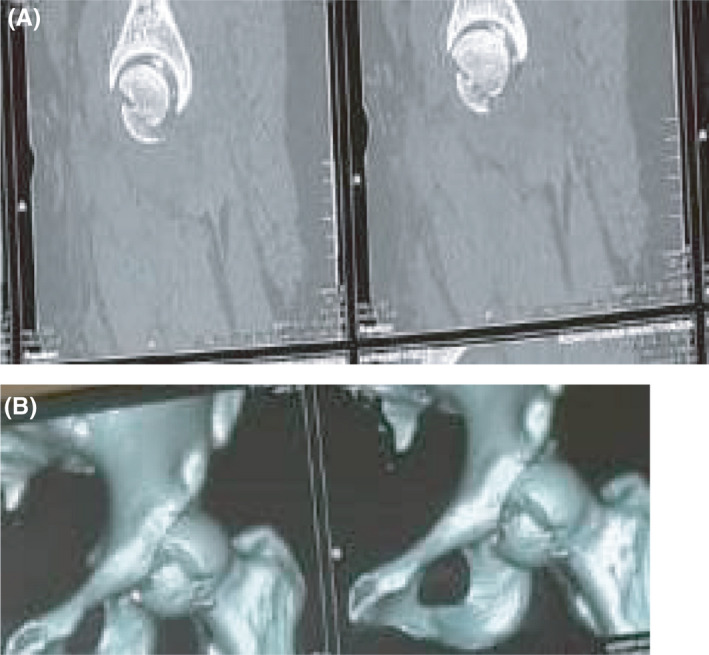
A and B, Postreduction axial CT scan (A) of the pelvis showing fracture of the femoral head with a nonanatomical reduction of the fragment and 3D reconstruction (B) showing femoral head fracture inferior to the fovea centralis (Pipkin I)

An emergent closed reduction under general anesthesia was done within 20 minutes of presentation to the emergency department, but about 3 hours from the time of the injury. There was no associated neurovascular deficit before or after the reduction. Following the reduction, the hip was found to be relatively unstable. Postreduction pelvic X‐ray showed an incongruent and widened hip joint (Figure 2). A CT scan of the pelvis with 3D reconstruction showed a large femoral head fragment inferior to the fovea centralis that was not anatomically reduced (Figure 3A and B). An open anatomical reduction and internal fixation using safe surgical hip dislocation as described by Ganz ^10^ was done 4 days after the initial. The delay was as a result of unavailability of Herbert screws at the time of presentation.

The patient was placed in the right decubitus position and the incision centered over the left greater trochanter (GT), extended 5 cm above and 7 cm below the trochanter. This was followed by splitting of the fascia lata. The leg was internally rotated, bringing the posterior border of the gluteus medius into focus. An incision extending from the posterosuperior margin of the GT to the posterior border of the ridge of the vastus lateralis was done. An oscillating saw was then used to osteotomized the GT (thickness of about 1.5 cm) along the line described above. The GT together with the attached vastus lateralis was mobilized anteriorly after releasing it along the posterior border to the midportion of the gluteus maximus tendon. The vastus lateralis and intermedius were elevated from the lateral and anterior aspect of the femur with the leg flexed and externally rotated. The posterior border of the gluteus medius was retracted anterosuperiorly to reveal the piriformis tendon. The inferior margin of the gluteus minimus was then gently dissected from the piriformis and the joint capsule. The flap involving the gluteus minimus was retracted anterosuperiorly, and with further flexion and external rotation of the hip, the capsule was visualized. A T‐shaped anterior capsulotomy was done. The hip was dislocated by flexion and external rotation of the leg. This allowed inspection of the femoral head and acetabulum.

**FIGURE 4:**
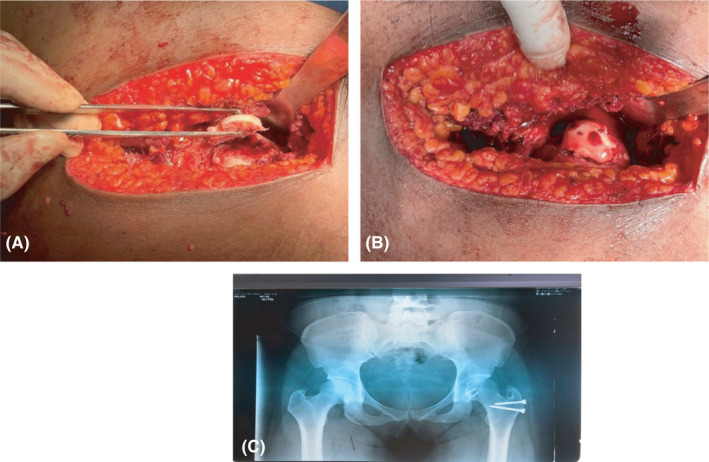
A‐C, Intraoperative image showing the large femoral head fragment (A) and reduced fragment (B) fixed with two 2.7 mm Herbert screws subchondrally and (C) postoperative pelvic X‐ray showing screw fixation of the greater trochanter

Intraoperatively, the fragment was found to be viable (viability of the femoral head was confirmed by observing bleeding from the fragment following perforation using a 1.6 mm Kirschner wire); hence, anatomical reduction was done followed by fixation of the fragment using two 2.7 mm subchondral headless cannulated screws (Herbert screws) on the posterio‐inferior aspect of the head (Figure 4A and B). The labrum was found on inspection to be torn and was repaired using Vicryl 2 suture. The capsule was closed with Vicryl 2. The greater trochanter was fixed using two 3.5 mm cortical screws (Figure 4C).

**FIGURE 5:**
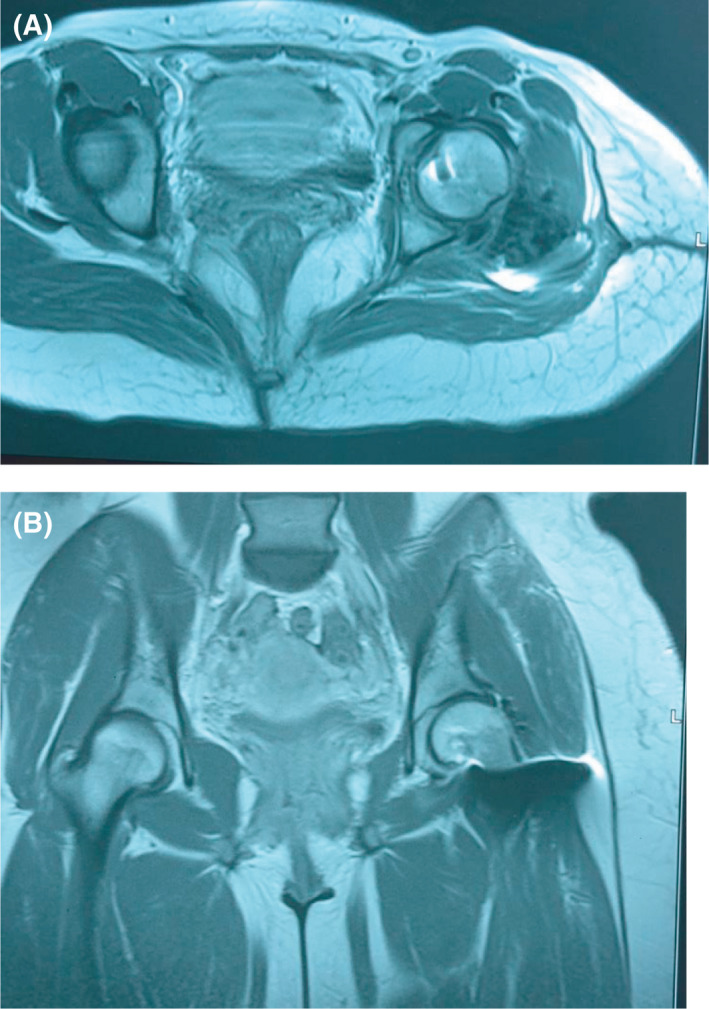
A and B, Axial (A) and sagittal CT scans (B) as well as AP pelvic X‐ray at 1 year postinjury showing healed fracture with no evidence of avascular necrosis

Postoperatively, the patient was allowed to touch weight bearing on crutches for 8 weeks followed by full weight bearing. She was put on 25 mg indomethacin (trice daily for a month) as a prophylaxis against heterotopic ossification. At 1‐year follow‐up, she had a painless hip with a full range of motion and there was no evidence of AVN of the femoral head (Figure 5A and B) or heterotopic ossification.

## 3 DISCUSSION

The most important determinant of optimum outcome is the time between the dislocation and reduction of the hip joint. Epstein et al^7^ recommended that all traumatic hip dislocations should be managed as surgical emergencies and multiple attempts at closed reduction should be avoided to minimize the risk of AVN which has an incidence between 8% and 26%.^2^ According to Epstein,^12^ reduction within 24 hours lead to better outcome compared to when done after 24 hours. McMurtry et al^13^ further indicated that the risk of AVN of the femoral is small when reduction is done within 6 hours.

Pipkin I fractures can be managed either surgically or nonoperatively depending on the fragment size and surgical expertise. In general, surgical management is recommended for types I and II fractures with large fragments especially those located at the weight‐bearing region of the head as well as all types III and IV.^14^, ^15^ Achieving optimal results is dependent on obtaining an anatomical reduction of the fragments, which is difficult with closed reduction. Henle et al^9^ recommended surgical treatment to improve the reduction if the gap between the fragments were more than 2 mm as only 1 in 12 patients in their series had anatomical reduction following closed manipulation.

The best surgical approach for Pipkin fractures remains controversial.^1^, ^9^, ^10^ The Kocher‐Langenbeck, Smith‐Peterson, and Watson Jones approaches or percutaneous fixation after a successful reduction have been used in managing these injuries albeit with limited exposure of the femoral head and acetabulum.^7^, ^8^, ^12^ The classical Kocher‐Langenbeck (posterior) approach permits direct access to the injured hip and capsule. There is, however, a high risk of iatrogenic injury to the deep branch of the medial circumference femoral artery, leading to avascular necrosis and arthritis of the hip.^6^, ^16^ The femoral head can be safely dislocated using the Smith‐Petersen (anterior) approach. However, inspection of the acetabulum may be limited unless you detach the tensor fascia lata and gluteus medius from their origin.^10^ Swiontkowski et al^17^ compared the use of the anterior and posterior approaches in the management of Pipkin fractures in terms of blood supply to the femur and concluded that the anterior approach was much safer with respect to damage to the blood supply, operating time was shorter, less blood loss, and offered better visualization of the femoral head. There was, however, an increased risk of heterotopic ossification. Dislocation of the femoral head through the anterolateral or direct lateral approaches is also possible. It is, however, difficult to expose and visualize the acetabulum using these approaches.^18^, ^19^

Safe SHD has been increasingly used in addressing head of femur fractures despite its technical challenges.^10^, ^20^ It enables full access to the whole acetabulum and femoral head, facilitates the anatomical reduction of the fragments, and also helps identify any chondral, subchondral, or labral tears which may not be seen using the other approaches.^1^, ^2^, ^10^ Labral tears are found in up to 50% of patients and are associated with poor outcome.^2^ The functional outcome (in terms of pain and range of motion of the hip joint) of our patient was satisfactory probably because it was a Pipkin type I fracture. However, the choice of safe SHD may have contributed to this outcome as the approach minimizes the risk of vascular injury to the head and the already traumatized soft tissue.

## 4 CONCLUSION

This case demonstrates the efficacy of safe surgical hip dislocation in managing femoral head fractures. The type of treatment and surgical approach should be guided by the fracture type and the associated injuries. Irrespective of the method or surgical approach, however, we should always aim for anatomical reduction of the fragments while minimizing injury to the surrounding soft tissues.

## CONFLICT OF INTEREST

None declared.

## AUTHOR CONTRIBUTIONS

PKB, KTB, AA, AOA, EKA, and SG: performed the surgical procedure, followed up on the patient, and prepared the manuscript.

## ETHICAL APPROVAL

Written informed consent was obtained from the patient for publication of this case report and the associated images.

## Data Availability

Data sharing is not applicable to this article as no new data were created in this study.
